# Role of Spectrin in Endocytosis

**DOI:** 10.3390/cells11152459

**Published:** 2022-08-08

**Authors:** Donghai Li

**Affiliations:** State Key Laboratory of Pharmaceutical Biotechnology, School of Life Sciences, Nanjing University, Nanjing 210023, China; donghaili@nju.edu.cn

**Keywords:** cytoskeleton, endocytosis, evolution, spectrin

## Abstract

Cytoskeletal spectrin is found in (non)erythroid cells. Eukaryotic endocytosis takes place for internalizing cargos from extracellular milieu. The role of spectrin in endocytosis still remains poorly understood. Here, I summarize current knowledge of spectrin function, spectrin-based cytoskeleton and endocytosis of erythrocytes, and highlight how spectrin contributes to endocytosis and working models in different types of cells. From an evolutionary viewpoint, I discuss spectrin and endocytosis in a range of organisms, particularly in plants and yeast where spectrin is absent. Together, the role of spectrin in endocytosis is related to its post-translational modification, movement/rearrangement, elimination (by proteases) and meshwork fencing.

## 1. Introduction

**Figure 1:**
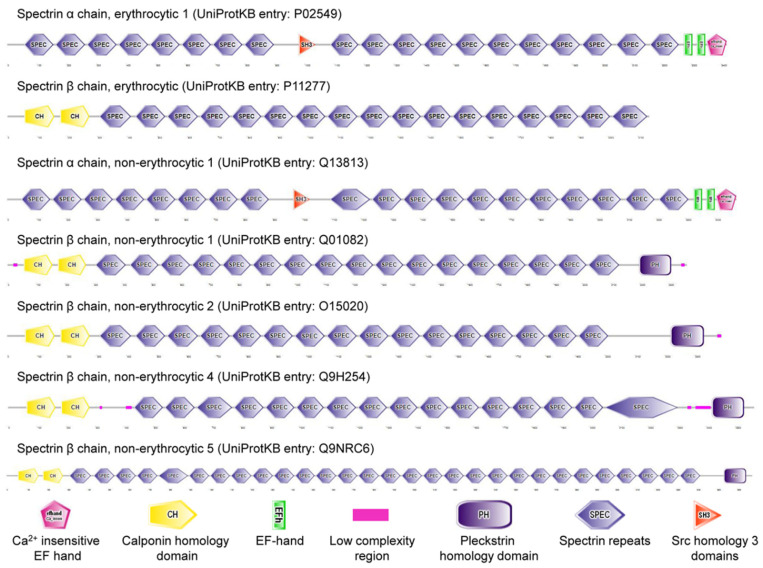
Spectrins in *Homo Sapiens*.

Cytoskeletal spectrin (Latin: derived from ghosts) originally discovered in erythrocytes (commonly known as red blood cells, RBCs) is expressed in erythroid and nonerythroid cells. Spectrin, the main component of the RBC cortex, is a long, flexible, rod-shaped heterodimeric protein (100 nm in length), and further self-associates into a heterotetramer. UniProt KB entries of human erythrocyte spectrin are P02549 (α spectrin, αSp; 2419 amino acids) and P11277 (β spectrin, βSp; 2137 amino acids) [1]. There are seven mammalian spectrin genes (two α-genes and five β-genes, Figure 1). Three spectrin subunits (α, β_G_, and β_H_) are present in invertebrate cells. Some isoforms of each spectrin gene are produced by alternative pre-mRNA processing (or, theoretically, alternative promoter use) and Arabic numerals are added after the symbol Σ to denote subtype (e.g., αSpIIΣ1, αSpIIΣ2, αSpIIΣ3, αSpIIΣ4, βSpIVΣ1, βSpIVΣ2, βSpIVΣ3, βSpIVΣ4) [2,3]. These isoforms are located in diverse cellular compartments, including membrane, Golgi apparatus, endoplasmic reticulum, vesicles, and nucleus [4]. The membrane skeleton located underneath plasma membrane of mammalian erythrocytes interacts with the lipid bilayer by membrane proteins. The interactions between the membrane skeleton and membrane proteins are called as vertical interactions, and include ankyrin-based complex and junctional complex, while the interactions between the components of membrane skeleton itself are known as horizontal interactions [5]. Ankyrins, well-known adaptor proteins, were first identified as a partner of spectrin in the plasma membrane of erythrocytes. It is established that spectrin and ankyrin are required for specialized membrane domains in various types of cells [6]. Spectrin integrates into a quasi-hexagonal lattice of erythrocyte membrane skeleton [7,8,9], while it appears to organize into an erythrocyte-like, pentagonal or hexagonal lattice in *Drosophila* motoneuron axons near neuromuscular junctions [10]. Interestingly, spectrin locates at a quasi-one-dimensional, periodic, ladder-like structure in axons of vertebrate brain [11].

In addition to its function as a cytoskeletal scaffolding protein, erythrocyte spectrin constitutes a part of ATPase, and exhibits chaperone and chimeric E2/E3 ubiquitin conjugating/ligating activities [12,13,14,15,16,17]. Spectrin plays a key role in cell processes and signaling, including maintenance of stability and structure of cell membrane and cell shape, cell adhesion, cell spreading, cell cycle, apoptosis, and trafficking of proteins, vesicles and organelles [4,18,19,20,21,22,23,24,25,26]. Nonerythroid αSpII located in nuclei distributes in the peripheral nucleoskeleton and inner nucleoskeleton and has structural and non-structural roles such as nuclear architecture and organization, and DNA repair [27]. βSpIII is a major component of Golgi and vesicular membrane skeletons for maintaining the structure of Golgi apparatus and orchestrating protein traffic in the secretory pathway [4,28,29]. Spectrin in *Drosophila melanogaster* or in *Caenorhabditis elegans* is suggested to be important for survival and normal development [30]. Dysfunction of spectrin is implicated in human diseases such as hemolytic anemia (e.g., two common inherited RBC membrane disorders: hereditary elliptocytosis and hereditary spherocytosis), nervous system diseases, and cancer [31]. Dysfunctions of horizontal and vertical interactions may lead to hereditary elliptocytosis and spherocytosis, respectively [32,33,34]. A significant deficiency of αIISp in nuclei is identified in a disorder such as Fanconi anemia (FA), but the relationship between a deficiency of αIISp and FA-patient-involved congenital abnormalities is ambiguous.

RBCs are a potential drug delivery system and entrap compounds by endocytosis [35,36,37,38,39,40,41]. Eukaryotic endocytosis relates to nutrient uptake, receptor internalization, antigen presentation and synaptic transmission. Dysfunction of endocytic pathway has been reported in human diseases such as neurodegenerative diseases, cancer and virus infections [42,43]. The concept of endocytosis could date back to the end of the 19th century. Élie Metchnikoff was the first to identify phagocytes as a part of immune system [44]. Christian de Duve (1917-2013), the discoverer of lysosomes (1955), coined the terms “endocytosis” (1963) (new term for athrocytosis), “phagocytosis”, and “autophagy” (1963) to describe pathways bringing substrates for digestion in lysosomes [42,44,45]. Endocytosis includes phagocytosis (cell eating), which depicts ingestion of large particles such as bacteria and plays specialized roles, and pinocytosis (cell drinking) discovered by 1930, which depicts uptake of fluids or macromolecules in small vesicles [46,47]. With respect to pinocytosis, the best-characterized clathrin-mediated endocytosis (CME, also referred to as receptor-mediated endocytosis, RME) is used for selective uptake of specific macromolecules. The present model of CME is as follows: macromolecules (ligands) to be endocytosed first bind to specific receptors which in turn cluster in specialized regions of plasma membrane where clathrin-coated membrane invaginates, called clathrin-coated pits; these pits bud from plasma membrane to form small clathrin-coated and receptor/ligand-containing vesicles; the coats are shed from the clathrin-coated vesicles and then uncoated vesicles fuse with early endosomes; finally, endocytic contents in the early endosomes are sorted to lysosomes for degradation or recycling to the plasma membrane [46]. These endocytic proteins of CME can be divided into functional modules, including clathrin coat module, actin module formed by polymerization of a network of actin filaments at the endocytic site, scission module, and uncoating module for driving the disassembly of endocytic machinery [48].

## 2. Erythroid Cells and Endocytosis

Endocytosis is seen in erythrocytes of splenectomized people or when patients are functionally asplenic as in sickle cell anemia [49]. It is observed that endocytosis occurs in intact erythrocytes and ghosts. A “ghost” is the membrane isolated from erythrocytes subjected to hemolysis/haemolysis. It is subdivided into resealed ghost and white ghost (hemoglobin/haemoglobin-free) based on severity of hemolysis and hemoglobin removal. These ghosts can be utilized to investigate physiological and biochemical properties of erythrocyte membrane [50]. In 1968, pinocytosis was observed by electron microscopy in erythrocyte ghosts (free of most of residual hemoglobin) prepared from cow, pig and rabbit blood, and required ATP and was not induced by imposition of external osmotic pressure [51,52]. Endocytic vacuole formation of human erythrocyte (sealed) ghosts requires Mg^2+^ and ATP, and relatively specific energization by ATP is important [37,53]. Thus, this type of endocytosis is considered as an energy-requiring process [54]. In addition, endocytosis of ghosts was induced in the absence of added Mg^2+^-ATP by treatment with triton X-100 or sucrose, suggesting that ultrastructural changes (ghost membranes became expanded and many small vesicles were seen within each parent ghost) of ghosts membranes related to triton X−100 or sucrose had no connection with hydrolysis of ATP [55,56]. Taken together, two forms of endocytosis of erythrocyte ghosts were identified, i.e., “nonenergized” (hypotonicity, EDTA or trypsin addition) and “energized” (Mg^2+^-ATP addition) endocytosis. 

The effect of primaquine, chlorpromazine or vinblastine on intact erythrocytes indicated that drug-induced endocytosis was not necessarily an energy-dependent process [57]. In 1969, the first evidence that vacuole formation was induced by primaquine in intact erythrocytes and ghosts was reported, and the mechanism of vacuole-formation-involved membrane internalization was proposed [58]. According to studies of hypotonic dialysis-prepared human erythrocyte carriers, methotrexate was taken up primarily by endocytosis and secondarily by passive diffusion, and did not induce endocytic activity [41]. Notably, there are different characteristics in neonatal and adult RBCs such as endocytic activity. Compared with those in adult RBCs (2.6%), endocytic vacuoles appear frequently and spontaneously in neonatal RBCs (a mean of 47.2% for premature infants, 24.3% for term infants) [59]. Endocytosis exists in membrane-active drug-induced adult RBCs [37,58], but RME is lacking [60,61,62]. Only neonatal puckered RBCs underwent RME and were identified as motile R-1 reticulocytes [60,61,62,63]. Myosin found in neonatal and adult erythrocytes had different amounts and distribution, which could cause some of the unusual properties of neonatal RBCs [64]. A comparative study of transferrin RME and drug-induced endocytosis in human neonatal and adult RBCs demonstrated that energy requirement, response to inhibitors and morphologic concomitants contributed to characteristic difference between RME of motile reticulocytes in neonatal RBCs and drug-induced endocytosis [62]. A separate study compared spontaneous endocytosis in human neonatal and adult RBCs based on the rate and quantity of vacuoles and the shape [65]. The results indicated that spontaneous endocytosis was different from drug-induced endocytosis and transferrin RME. An explanation for the increase in spontaneous endocytosis in cord RBC seen in vivo is an immaturity of neonatal macrophage-pitting process [65]. ATP hydrolysis is required for certain forms of amphipathic drug (primaquine, chlorpromazine and vinblastine)-induced stomatocytosis and endocytosis in intact RBCs and endocytosis in white ghosts [66]. A review summarized drug-induced endocytosis and entrapment in RBCs and ghosts [49]. The human erythroleukemia cell line, K562, which has endocytic activity, is a predecessor of erythroblasts (a precursor of erythrocytes). Therefore, K562 cells were also used to study transferrin RME and iron uptake, and iterative endocytosis of transferrin for explaining iron-transferrin release and high efficiency of iron-uptake process [67,68]. 

Endocytosis in RBCs with membrane disorders has also been reported. Drug-induced vacuole formation was impaired in RBCs with hereditary spherocytosis, but vacuole formation in (sealed) ghosts was normal [69]. Hereditary pyropoikilocytosis (HPP) is a subtype of hereditary elliptocytosis. Drug-induced endocytosis increased in neonatal RBCs with HPP [70]. Additional studies are needed to elucidate mechanism of (spectrin-mediated) endocytosis in RBCs with diseases.

## 3. Erythroid Spectrin

Actin cytoskeleton is an integral part of cell cortex, and there is growing evidence that filamentous actin (F-actin) plays a direct role in endocytic events [71]. Spectrin, a actin cross-linking protein, was also investigated for its endocytosis-related role. In 1978, the role of erythroid spectrin in endocytosis was evaluated in white ghosts [56]. During “nonenergized” and “energized” endocytosis, endocytic vacuoles were spectrin-free and anti-spectrin antibody pretreatment inhibited endocytosis of white ghosts [56,72]. A hypothesis was proposed that endocytosis of white ghosts required formation of spectrin-free domains and that manipulations of limiting spectrin movement would block endocytosis [56]. Due to the effect of the spectrin–actin lattice on erythrocytic shape changes and inhibition of actin polymerization by spectrin, a regional release of spectrin may allow actin polymerization [56,73,74]. After that, transmission electron microscopy, and radioiodinated and ferritin-tagged anti-spectrin antibodies were used. Pretreatment of ghosts with alkaline phosphatase blocked endocytosis and creation of spectrin-free areas [75]. The literature described a detailed model of Mg^2+^-ATP/trypsin/EDTA-induced endocytosis. Initially, agent-induced ghost endocytosis was involved in phosphorylated spectrin; loose spectrin interactions were eliminated to form spectrin-free domains separated by residual spectrin clusters; subsequent invagination and membrane fusion occurred in the spectrin-free zones [75]. Similar to the results of erythrocyte ghosts, concanavalin A (Con A, an artificial ligand)-induced domains of receptor mobility and endocytosis in plasma membranes of neonatal erythrocytes and reticulocytes were also spectrin-depleted [61]. It was hypothesized that there were (induced) specialized discrete domains in plasma membranes of neonatal cells, where Con A receptors were laterally mobile, whereas in the remaining (and predominant) part of plasma membranes, Con A receptors were immobile. Such mobile domains are spectrin-free. Clustering of Con A receptors was required for such endocytosis, with clustered receptor-containing membrane invaginations [60]. Clustering of receptors and absence or clearing of spectrin from the clustered region might alone be responsible for a progressive invagination of the membrane. Other mechanochemical factors might also be involved, particularly for scission of invagination of the membrane to form vesicles. These regions of invagination membrane formed a “collar” around the spectrin-free domain to conduct a contractile activity. These spectrin-free domains were significantly, but not completely, eliminated during maturation of neonatal reticulocytes to erythrocytes [61]. Inside-out vesicles and endocytic vacuoles produced in white ghosts and exocytic vesicles produced from intact RBCs are variably depleted of spectrin and actin [72]. A percolation model was developed to successfully understand the main results of endocytosis [76]. Notably, human erythrocyte membrane vesicles produced by shearing were different from spectrin-depleted endocytic vacuoles and retained high concentrations of spectrin [77]. A simulation analysis investigated the effect of the spectrin-actin membrane skeleton on internalization of nanoparticles in RBCs [78]. The results showed that spectrin-actin membrane skeleton induced the effect of preventing nanoparticles from internalization. Successful internalization of nanoparticles provided two possibilities: a smaller size of nanoparticles than the dimensions of skeleton meshes and rather weak skeleton tension for moving inward for nanoparticles internalization.

As described above, there were specific differences between neonatal and adult RBCs such as endocytosis. Surprisingly, neonatal and adult RBCs showed no differences in protein composition (e.g., spectrin), and had equivalent quantity of spectrin dimer (5 ± 2%) and similar tryptic peptide pattern of spectrin [79,80]. Dissociated dimer pairs, single dimers and missing tetramers of spectrin do not serve as a barrier to protein diffusion, but tetramers do [76,81]. Presumably, spectrin rearrangement could differentially appear in neonatal and adult RBCs [75].

Interestingly, rabbit reticulocytes, but not rabbit erythrocytes, absorbed Con A by endocytosis to a limited extent, different from pinocytosis-observed rabbit erythrocyte ghosts mentioned above. Also, the extent of endocytosis correlated with the maturation of reticulocytes. The results were consistent with the proposal on Con A-induced domains of receptor mobility and endocytosis, and further explained as progressive elimination of gaps or imperfections in the spectrin network [76,82].

## 4. Nonerythroid Spectrin

Endocytic processes have been identified and characterized in other types of cells such as epithelial cells, fibroblasts, neurons, sertoli cells, endothelial cells and human podocytes. Sertoli cells, recognized as phagocytic/pinocytic/secretory cells and the main differentiating cell type within developing testis, are a model system for studying cytoskeleton/junction interrelationships. Intermediate filaments in most epithelia are of keratin type, whereas those in mature Sertoli cells are of vimentin type [83]. Sertoli cells can perform endocytosis such as RME. Linkage between cytoskeletal proteins α spectrin and ZO−1 and gap junction protein connexin-43 in cardiac myocytes and role of actin during gap junction membrane endocytosis of rabbit granulosa cells indicated that spectrin/connexin−43/ZO−1/actin complex could function in gap junction plaque endocytosis of Sertoli cells [84,85,86]. In invertebrates, β-spectrin (17 spectrin repeats)/β_H_-spectrin (30 spectrin repeats) isoforms dimerize with a common α subunit, and β-spectrin and β_H_-spectrin are confined to basolateral domain and apical domain in epithelia, respectively. The potential negative relationship between β_H_-spectrin and endocytosis has been previously proposed in epithelial photoreceptor cells of *Drosophila* [87]. Additional study of transgenic *Drosophila* showed that β_H_-spectrin inhibited endocytosis and its C-terminal domain interacted with endocytic machinery [88]. On the basis of these data, Crumbs, a central regulator of epithelial apical-basal polarity in *Drosophila*, could recruit β_H_-spectrin to downregulate endocytosis [87,88]. However, a causal relationship between β_H_-spectrin inhibition of endocytosis and cell internalization should be further confirmed. In other systems, nonerythroid spectrin-based endocytosis has also been studied and relative results and potential models are summarized as follows.

### 4.1. Epithelial Cells

Brush border of intestinal epithelium is a model system for studying cortical cytoskeleton. The apical brush border is a highly specialized apical membrane developed by absorptive epithelium to facilitate exchange between the extracellular milieu and cells. The brush border is conserved across vertebrate and invertebrate species and can be divided into three subdomains, i.e., microvilli, exocytic/endocytic zone at the base of the microvilli, and terminal web in the apical cytoplasm [89,90]. Microvilli (1–2 μm long) contain a core of bundled actin microfilaments. Actin filaments terminate in “rootlets” that bind to actin, spectrin, MYO2 and cytokeratins to construct a terminal web [90,91]. Coated pits and endocytic machinery were identified in the specialized intermicrovillar domain of the brush border of proximal tubule epithelia cells. This was less apparent in enterocytes [90]. In enterocytes, RME was regulated by MYO6, which was located to the subapical terminal web region and tethered the plasma membrane to bundled actin filaments [92]. Brush-border “fanning”, a condition caused by interferon-γ-induced myosin II-dependent contraction of the terminal web, allowed bacteria access to the base of microvilli, where bacteria were internalized by lipid raft-mediated endocytosis [92]. The spectrin-actin membrane skeleton in the terminal web is not so closely juxtaposed to the plasma membrane endocytoic zone at the base of the microvilli so that a hypothesis is suggested that spectrin removal is not required for endocytosis. Many vesicles are seen in the terminal web and these associate with spectrin during their traverse. Epistasis tests indicated that *Drosophila* brush border β_H_-spectrin was required during endocytosis after dynamin (scission-related proteins) and before Rab5 (early endosome marker)-mediated endosome activities. A model was proposed for the role of brush-border spectrin in the recycling pathway. Endocytic vesicles must go through spectrin-contained terminal web before proceeding to early endosomes. β_H_-spectrin “primes” protein on vesicles in the terminal web for correct sorting decisions at the early endosome [89]. Another model system used for investigating pinocytosis at the porcine intestinal brush border of post-weaned animals is organ-cultured mucosal explants. Alexa hydrazide, a small polar probe, was taken up into subapical early endosomes located in vicinity of spectrin within the terminal web by constitutive pinocytosis, which was not involved in lipid-raft microdomains and REM induced by cholera toxin B subunit [93]. 

### 4.2. Fibroblasts

Two in vitro cell systems were used to reconstitute clathrin-coated pit budding and study how endocytic vesicles form, i.e., perforated A431 cells and purified human fibroblast membrane. The fibroblast plasma membrane contains low-density lipoprotein (LDL) receptors clustered in LDL-induced clathrin-coated pits. The latter system suggested that fibroblasts produced at least two types of coated pits, one of which required annexin VI-dependent activation of a cysteine protease to disconnect the clathrin lattice from the spectrin membrane cytoskeleton accompanied by the loss of ~50% of spectrin from the plasma membrane, the other of which was produced due to cell adaption to the presence of cysteine protease inhibitor (*N*-acetyl-leucyl-leucyl-norleucinal, ALLN) and budded independently of annexin VI and spectrin removal [94]. Also, the endosomes formed from the second coated pits showed a distinctly different trafficking pattern. Calpastatin, an endogenous calpain inhibitor, had a similar inhibiting effect to ALLN. These results also suggested that activity of calpain I-like protease was linked to remodeling of the spectrin-based cytoskeleton during endocytic budding [94]. The D4 and D34 (domains 3 and 4) peptides of ankyrin inhibited budding from membranes prepared from untreated but not ALLN-treated SV589 fibroblasts [95]. The D4 and D34 peptides of ankyrin also blocked spectrin removal, but coated pit budding of ALLN-treated fibroblast was not accompanied by spectrin removal [94,95]. A study of SH3 domains and endocytosis showed that unlike amphiphysin SH3 domain that showed a potent blockage in RME, the SH3 domain of spectrin failed to exert any effect on endocytosis of transferrin uptake in COS−7 fibroblasts [96].

To investigate cytoskeletal assembly and cell-driven mechanoresponse, fibroblast spreading is considered as a stereotypical model. A relative study reported clathrin-coated pit distribution and dynamics of immortalized mouse embryonic fibroblasts during cell mechanoresponse to elucidate whether spectrin was implicated in RME [97]. Imaging analysis of fixed and live specimens and unperturbed fibroblast lifetime analysis of adaptor protein complex 2 (AP2, clathrin-adaptor protein) pits was performed. The results indicated that AP2 pits were fenced by a spectrin meshwork and supported a fencing mechanism by βII-spectrin where local endocytic capacity would be influenced by meshwork concentration and condensation. In regulation of endocytosis, membrane-binding sites of spectrin dominated, but actin-binding sites of spectrin were more essential during the actin-cytoskeleton-linked mobility processes.

### 4.3. Neurons

From a cell-biology-based standpoint, neurons are similar to epithelial cells in the specialization of plasma membrane into domains, but are different in the spatial separation of these domains [98]. Neurons and other cell types have the same fundamentals of endosomal recycling and degradation. Endocytosis in neurons can be broadly classified as clathrin-dependent mechanism and clathrin-independent mechanism such as macropinocytosis. Two spectrin-related examples are presented, including close homolog of L1 (CHL1, also referred to as CALL) and α-amino-3-hydroxy-5-methyl-4-isoxazolepropionic acid receptors (AMPARs). They are both endocytosed via clathrin-dependent mechanism [99,100].

CHL1, a cell adhesion molecule, plays a dual role by either promoting or inhibiting neuritogenesis. Cell-surface-bound CHL1 antibody uptake in cultured mouse hippocampal neurons was analyzed to measure CHL1 endocytosis. The results showed that CHL1 endocytosis depended on the integrity of lipid rafts and local reorganization of the cytoskeleton. Antibody-induced endocytosis of CHL1 was enhanced by βII spectrin expression knockdown by siRNA interference. Therefore, spectrin meshwork participated in removal of CHL1 from neuronal surface by endocytosis [101]. 

AMPARs mediate fast excitatory transmission in the central nervous system and a significant part of AMPARs is attached to the postsynaptic cytoskeleton. GluR1 is a subunit of AMPARs. GluR1/antibody complex uptake in cultured mouse hippocampal neurons was used to investigate AMPAR endocytosis. βI spectrin knockdown by siRNA interference or engineered microRNA resulted in an increased GluR1 internalization index, indicating that spectrin meshwork disassembly increased AMPAR internalization [102].

## 5. Evolution of Spectrin and Endocytosis

**Figure 2:**
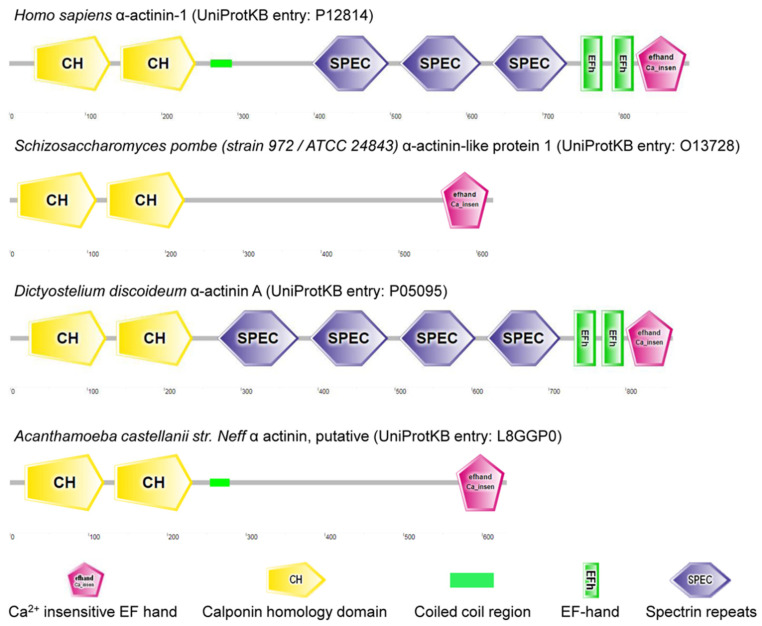
α-actinin homologs in *Homo sapiens*, *Schizosaccharomyces pombe*, *Dictyostelium discoideum* and *Acanthamoeba castellanii str. Neff*.

Spectrin evolves with the metazoans [103]. It is suggested that α and β spectrins evolve from a homodimeric α-actinin-like precursor polypeptide [30]. α-actinin is a cytoskeletal actin-binding protein and a member of spectrin superfamily arising from a common ancestral α-actinin gene through duplications and rearrangements (Figure 2) [104]. So far, no spectrin/actinin has been annotated in plant genomes such as *Zea mays*, *Oryza sativa* and *Solanum lycopersicum*. Spectrin is also absent from *Saccharomyces cerevisiae S288C* (budding yeast) and *Schizosaccharomyces pombe* (fission yeast) genomes, but α-actinin-like protein 1 (gene: *ain1*) is found in *Schizosaccharomyces pombe*. Of note, α-actinin-like protein 1 (UniProtKB entry: O13728) has two calponin homology domains and a Ca^2+^ insensitive EF hand without a spectrin repeat, as predicted by SMART (Figure 2) [105]. Candidates for prototypic spectrin have been characterized in *Dictyostelium* and *Acanthamoeba*, and no spectrin sequence appears in genomes of *Dictyostelium discoideum AX4* or *Acanthamoeba castellanii str. Neff* (Figure 2). 

It is known that endocytosis occurs for internalizing materials from the extracellular milieu in eukaryotic cells [106]. Endocytosis in yeast, and animal and plant systems has been established [71,107]. Although endocytosis was considered theoretically impossible in plants because of high turgor pressure, subsequent studies demonstrated that plants possessed the ability for endocytosis required for nutrient uptake, immune response, turnover of plasma membrane proteins, and hormone and stress signaling [107,108]. In plants, CME is the major mechanism and its model has been proposed [109]. Starting from endocytic vesicle formation, AP2 binds to phosphatidylinositol−4,5-bisphosphate, which is also an lipid-binding partner of endocytic proteins in animals, to associate with the plasma membrane. Clathrin and accessory proteins are recruited by membrane-associated AP2, which in turn binds to endocytic cargo proteins (e.g., plasma membrane-resident receptors and transporters). Accessory proteins help AP2 continue to recruit clathrin. Clathrin polymerizes and forms a clathrin coat around formed clathrin-coated pits. When clathrin-coated vesicles mature, dynamin-related protein is recruited at the neck of the vesicles to release the vesicle from the plasma membrane. Once vesicles are pinched off, coated components are disassembled and endocytic vesicles are released into the cytosol. The cortical actin cytoskeleton is implicated in CME [109]. However, the role of actin and microtubule cytoskeletons in CME still needs to be explored. 

Yeast is a powerful model system for investigating endocytosis. Many molecular mechanisms of CME are conserved from yeast to mammals and were first revealed in yeast. The difference between yeast and mammalian endocytic mechanisms has been summarized, including requirements for clathrin, actin polymerization and clathrin structures [110]. A model for the early endocytic pathway in budding yeast links recruitment of actin to the endocytic process. In this model, coat proteins are recruited to endocytic sites of the plasma membrane. The plasma membrane is deformed to cause invagination, and then actin is recruited to help the later stages of vesicle formation and the rapid movement of vesicles away from the plasma membrane [111,112]. Actively growing budding yeast contains three actin structures, i.e., cortical actin patches, actin cables and a contractile actin ring. The cortical actin patches are thought to represent sites of endocytosis [112].

Spectrin does not participate in endocytosis of plants and yeast due to its absence, and thus the actin cytoskeleton may play an essential role in endocytosis of these organisms. In budding yeast, α-actinin-like protein 1 has been shown to bind and bundle F-actin [113], but it remains undiscovered whether it replaces spectrin to play a role in endocytosis.

## 6. Conclusions

In summary, the proteins and molecular mechanisms involved in endocytosis have been largely proposed, but it remains unknown how different endocytic components are coordinated and there is, as yet, no detailed spectrin-related working model [48]. For example, ATP was required for CME in mammalian cells, but mitochondrial uncouplers inhibited CME largely through cytoplasmic acidification, but not through energy depletion or dissipation of membrane potential, suggesting that ATP requirements were different in various eukaryotic systems [114]. Undoubtedly, spectrin plays a role in endocytosis and has distinct mechanisms, including its phosphorylation, movement/rearrangement, elimination (by proteases) and meshwork fencing, in various cell types. However, it still remains unclear how spectrin functions in endocytic pathways; in-depth study is therefore required for better understanding of the role and dynamics of spectrin in endocytosis in endocytic systems.

## Data Availability

Not applicable.

## References

[1] UniProt Consortium (2021). UniProt: The universal protein knowledgebase in 2021. Nucleic Acids Res..

[2] Goodman S.R., Chapa R.P., E Zimmer W. (2015). Spectrin’s chimeric E2/E3 enzymatic activity. Exp. Biol. Med..

[3] Winkelmann J.C., Forget B.G. (1993). Erythroid and nonerythroid spectrins. Blood.

[4] Machnicka B., Grochowalska R., Bogusławska D.M., Sikorski A.F., Lecomte M.C. (2011). Spectrin-based skeleton as an actor in cell signaling. Experientia.

[5] Tse W.T., Lux S.E. (1999). Red blood cell membrane disorders. Br. J. Haematol..

[6] Bennett V., Healy J. (2009). Membrane domains based on ankyrin and spectrin associated with cell-cell interactions. Cold Spring Harb. Perspect. Biol..

[7] Liu S.C., Derick L.H., Palek J. (1987). Visualization of the hexagonal lattice in the erythrocyte membrane skeleton. J. Cell Biol..

[8] Lux S.E. (2016). Anatomy of the red cell membrane skeleton: Unanswered questions. Blood.

[9] Pan L., Yan R., Li W., Xu K. (2018). Super-resolution microscopy reveals the native ultrastructure of the erythrocyte cytoskeleton. Cell Rep..

[10] Pielage J., Cheng L., Fetter R.D., Carlton P., Sedat J.W., Davis G.W. (2008). A presynaptic giant ankyrin stabilizes the NMJ through regulation of presynaptic microtubules and transsynaptic cell adhesion. Neuron.

[11] Xu K., Zhong G., Zhuang X. (2013). Actin, spectrin, and associated proteins form a periodic cytoskeletal structure in axons. Science.

[12] Hsu Y.J., Goodman S.R. (2005). Spectrin and ubiquitination: A review. Cell Mol. Biol..

[13] Chakrabarti A., Kelkar D., Chattopadhyay A. (2006). Spectrin organization and dynamics: New insights. Biosci. Rep..

[14] Bose D., Chakrabarti A. (2019). Localizing the chaperone activity of erythroid spectrin. Cytoskeleton.

[15] Bhattacharyya M., Ray S., Bhattacharya S., Chakrabarti A. (2004). Chaperone activity and Prodan binding at the self-associating domain of erythroid spectrin. J. Biol. Chem..

[16] Chakrabarti A., Bhattacharya S., Ray S., Bhattacharyya M. (2001). Binding of a denatured heme protein and ATP to erythroid spectrin. Biochem. Biophys. Res. Commun..

[17] Baskin G.S., Langdon R.G. (1981). A spectrin-dependent ATPase of the human erythrocyte membrane. J. Biol. Chem..

[18] Bennett V., Healy J. (2008). Organizing the fluid membrane bilayer: Diseases linked to spectrin and ankyrin. Trends Mol. Med..

[19] Wu S., Sangerman J., Li M., Brough G.H., Goodman S.R., Stevens T. (2001). Essential control of an endothelial cell ISOC by the spectrin membrane skeleton. J. Cell Biol..

[20] Zhang R., Zhang C., Zhao Q., Li D. (2013). Spectrin: Structure, function and disease. Sci. China Life Sci..

[21] De Matteis M., Morrow J. (2000). Spectrin tethers and mesh in the biosynthetic pathway. J. Cell Sci..

[22] Lee J.K., Coyne R.S., Dubreuil R.R., Goldstein L.S., Branton D. (1993). Cell shape and interaction defects in alpha-spectrin mutants of *Drosophila melanogaster*. J. Cell Biol..

[23] Metral S., Machnicka B., Bigot S., Colin Y., Dhermy D., Lecomte M.C. (2009). αII-spectrin is critical for cell adhesion and cell cycle. J. Biol. Chem..

[24] Martin S.J., O’Brien G.A., Nishioka W.K., McGahon A.J., Mahboubi A., Saido T.C., Green D.R. (1995). Proteolysis of fodrin (non-erythroid spectrin) during apoptosis. J. Biol. Chem..

[25] Nath R., Huggins M., Glantz S.B., Morrow J.S., McGinnis K., Nadimpalli R., Wang K.K. (2000). Development and characterization of antibodies specific to caspase-3-produced alpha II-spectrin 120 kDa breakdown product: Marker for neuronal apoptosis. Neurochem. Int..

[26] Devarajan P., Stabach P.R., De Matteis M.A., Morrow J.S. (1997). Na,K-ATPase transport from endoplasmic reticulum to Golgi requires the Golgi spectrin-ankyrin G119 skeleton in Madin Darby canine kidney cells. Proc. Natl. Acad. Sci. USA.

[27] Lambert M.W. (2018). Spectrin and its interacting partners in nuclear structure and function. Exp. Biol. Med..

[28] Stankewich M.C., Tse W.T., Peters L.L., Ch’Ng Y., John K.M., Stabach P.R., Devarajan P., Morrow J.S., Lux S.E. (1998). A widely expressed βIII spectrin associated with Golgi and cytoplasmic vesicles. Proc. Natl. Acad. Sci. USA.

[29] Gu F., Crump C., Thomas G. (2001). Trans-Golgi network sorting. Experientia.

[30] Bennett V., Baines A. (2001). Spectrin and ankyrin-based pathways: Metazoan inventions for integrating cells into tissues. Physiol. Rev..

[31] Li S., Liu T., Li K., Bai X., Xi K., Chai X., Mi L., Li J. (2021). Spectrins and human diseases. Transl. Res..

[32] Fowler V.M. (2013). The human erythrocyte plasma membrane: A Rosetta Stone for decoding membrane-cytoskeleton structure. Curr. Top. Membr..

[33] Narla J., Mohandas N. (2017). Red cell membrane disorders. Int. J. Lab. Hematol..

[34] Mohandas N., Gallagher P.G. (2008). Red cell membrane: Past, present, and future. Blood.

[35] Villa C.H., Anselmo A., Mitragotri S., Muzykantov V. (2016). Red blood cells: Supercarriers for drugs, biologicals, and nanoparticles and inspiration for advanced delivery systems. Adv. Drug Deliv. Rev..

[36] Li Y., Raza F., Liu Y., Wei Y., Rong R., Zheng M., Yuan W., Su J., Qiu M. (2021). Clinical progress and advanced research of red blood cells based drug delivery system. Biomaterials.

[37] Ben-Bassat I., Bensch K.G., Schrier S.L. (1972). Drug-induced erythrocyte membrane internalization. J. Clin. Investig..

[38] Schrier S.L., Hardy B., Bensch K.G. (1979). Endocytosis in erythrocytes and their ghosts. Prog. Clin. Biol. Res..

[39] Koleva L., Bovt E., Ataullakhanov F., Sinauridze E. (2020). Erythrocytes as carriers: From drug delivery to biosensors. Pharmaceutics.

[40] Ihler G.M., Tsang H.C.-W. (1987). Hypotonic hemolysis methods for entrapment of agents in resealed erythrocytes. Methods Enzymol..

[41] Kruse C.A., Mierau G.W., James G.T. (1989). Methotrexate loading of red cell carriers by osmotic stress and electric-pulse methods: Ultrastructural observations. Biotechnol. Appl. Biochem..

[42] Ellinger I., Pietschmann P. (2016). Endocytosis in health and disease—A thematic issue dedicated to Renate Fuchs. Wiener Medizinische Wochenschrift.

[43] Tagliatti E., Cortese K. (2022). Imaging endocytosis dynamics in health and disease. Membranes.

[44] Bainton D.F. (1981). The discovery of lysosomes. J. Cell Biol..

[45] Sabatini D.D., Adesnik M. (2013). Christian de Duve: Explorer of the cell who discovered new organelles by using a centrifuge. Proc. Natl. Acad. Sci. USA.

[46] Cooper G.M., Cooper G.M. (2000). Endocytosis. The Cell: A Molecular Approach.

[47] Lewis W.H. (1931). Pinocytosis. Bull. Johns Hopkins Hosp..

[48] Kaksonen M., Roux A. (2018). Mechanisms of clathrin-mediated endocytosis. Nat. Rev. Mol. Cell Biol..

[49] Schrier S.L. (1987). Drug-induced endocytosis and entrapment in red cells and ghosts. Methods Enzymol..

[50] Verma P.S., Agarwal V.K. (2005). Cell Biology, Genetics, Molecular Biology, Evolution and Ecology.

[51] Penniston J.T., Green D. (1968). The conformational basis of energy transformations in membrane systems: IV. Energized states and pinocytosis in erythrocyte ghosts. Arch. Biochem. Biophys..

[52] Marchesi V.T., Palade G.E. (1967). The localization of Mg-Na-K-activated adenosine triphosphatase on red cell ghost membranes. J. Cell Biol..

[53] Schrier S.L., Junga I., Seeger M. (1973). Vacuole formation in human erythrocyte ghosts. Exp. Biol. Med..

[54] Penniston J.T. (1972). Endocytosis by erythrocyte ghosts; dependence upon ATP hydrolysis. Arch. Biochem. Biophys..

[55] Katsumata Y., Asai J. (1972). Ultrastructural changes of erythrocyte ghosts having no connection with hydrolysis of ATP. Arch. Biochem. Biophys..

[56] Hardy B., Schrier S.L. (1978). The role of spectrin in erythrocyte ghost endocytosis. Biochem. Biophys. Res. Commun..

[57] Zarkowsky H., Rinehart J. (1979). Endocytosis in adenosine triphosphate-depleted erythrocytes. Biochim. Biophys. Acta Gen. Subj..

[58] Ginn F.L., Hochstein P., Trump B.F. (1969). Membrane alterations in hemolysis: Internalization of plasmalemma induced by primaquine. Science.

[59] Holroyde C.P., Oski F.A., Gardner F.H. (1969). The pocked erythrocyte. N. Engl. J. Med..

[60] Schekman R., Singer S.J. (1976). Clustering and endocytosis of membrane receptors can be induced in mature erythrocytes of neonatal but not adult humans. Proc. Natl. Acad. Sci. USA.

[61] Tokuyasu K., Schekman R., Singer S. (1979). Domains of receptor mobility and endocytosis in the membranes of neonatal human erythrocytes in the membranes of neonatal human erythrocytes and reticulocytes are deficient in spectrin. J. Cell Biol..

[62] Thatte H.S., Schrier S.L. (1988). Comparison of transferrin receptor-mediated endocytosis and drug-induced endocytosis in human neonatal and adult RBCs. Blood.

[63] Haberman S., Blanton P., Martin J. (1967). Some observations on the ABO antigen sites of the erythrocyte membranes of adults and newborn infants. J. Immunol..

[64] Colin F.C., Schrier S.L. (1991). Myosin content and distribution in human neonatal erythrocytes are different from adult erythrocytes. Blood.

[65] Colin F.C., Schrier S.L. (1991). Spontaneous endocytosis in human neonatal and adult red blood cells: Comparison to drug-induced endocytosis and to receptor-mediated endocytosis. Am. J. Hematol..

[66] Schrier S.L., Junga I., Ma L. (1986). Studies on the effect of vanadate on endocytosis and shape changes in human red blood cells and ghosts. Blood.

[67] van Renswoude J., Bridges K.R., Harford J.B., Klausner R.D. (1982). Receptor-mediated endocytosis of transferrin and the uptake of fe in K562 cells: Identification of a nonlysosomal acidic compartment. Proc. Natl. Acad. Sci. USA.

[68] Young S.P., Bomford A. (1994). Iterative endocytosis of transferrin by K562 cells. Biochem. J..

[69] Schrier S.L., Ben-Bassat I., Bensch K., Seeger M., Junga I. (1974). Erythrocyte membrane vacuole formation in hereditary spherocytosis. Br. J. Haematol..

[70] Matovcik L.M., Junga I.G., Schrier S.L. (1985). Drug-induced endocytosis of neonatal erythrocytes. Blood.

[71] Engqvist-Goldstein Å.E.Y., Drubin D.G. (2003). Actin assembly and endocytosis: From yeast to mammals. Annu. Rev. Cell. Dev. Biol..

[72] Schrier S.L., Junga I., Ma L. (1986). Endo- and exovesiculation and the structure of the human red cell membrane. J. Lab. Clin. Med..

[73] Tilney L.G., Detmers P. (1975). Actin in erythrocyte ghosts and its association with spectrin. Evidence for a nonfilamentous form of these two molecules in situ. J. Cell Biol..

[74] Lux S.E., John K.M., Karnovsky M.J. (1976). Irreversible deformation of the spectrin-actin lattice in irreversibly sickled cells. J. Clin. Investig..

[75] Hardy B., Bensch K.G., Schrier S.L. (1979). Spectrin rearrangement early in erythrocyte ghost endocytosis. J. Cell Biol..

[76] Saxton M. (1989). The spectrin network as a barrier to lateral diffusion in erythrocytes. A percolation analysis. Biophys. J..

[77] Schrier S.L., Junga I. (1980). Analysis of human erythrocyte membrane vesicles produced by shearing. J. Supramol. Struct..

[78] Gao X., Yue T., Tian F., Liu Z., Zhang X. (2017). Erythrocyte membrane skeleton inhibits nanoparticle endocytosis. AIP Adv..

[79] Shapiro D.L., Pasqualini P. (1978). Erythrocyte membrane proteins of premature and full-term newborn infants. Pediatr. Res..

[80] Lawler J., Liu S.C., Palek J., Prchal J. (1984). A molecular defect of spectrin in a subset of patients with hereditary elliptocytosis. Alterations in the alpha-subunit domain involved in spectrin self-association. J. Clin. Investig..

[81] Liu S.-C., Palek J. (1980). Spectrin tetramer–dimer equilibrium and the stability of erythrocyte membrane skeletons. Nature.

[82] Zweig S., Singer S.J. (1979). Concanavalin A-induced endocytosis in rabbit reticulocytes, and its decrease with reticulocyte maturation. J. Cell Biol..

[83] Vogl A.W., Vaid K.S., Guttman J.A. (2009). The Sertoli cell cytoskeleton. Adv. Exp. Med. Biol..

[84] Segretain D., Fiorini C., Decrouy X., Defamie N., Prat J., Pointis G. (2004). A proposed role for ZO-1 in targeting connexin 43 gap junctions to the endocytic pathway. Biochimie.

[85] Larsen W.J., Tung H.-N., Murray S.A., A Swenson C. (1979). Evidence for the participation of actin microfilaments and bristle coats in the internalization of gap junction membrane. J. Cell Biol..

[86] Toyofuku T., Yabuki M., Otsu K., Kuzuya T., Hori M., Tada M. (1998). Direct association of the gap junction protein connexin-43 with ZO-1 in cardiac myocytes. J. Biol. Chem..

[87] Pellikka M., Tanentzapf G., Pinto M., Smith C.T., McGlade C.J., Ready D.F., Tepass U. (2002). Crumbs, the *Drosophila* homologue of human CRB1/RP12, is essential for photoreceptor morphogenesis. Nature.

[88] Williams J.A., MacIver B., Klipfell E.A., Thomas G.H. (2004). The C-terminal domain of *Drosophila* β_Heavy_-spectrin exhibits autonomous membrane association and modulates membrane area. J. Cell Sci..

[89] Phillips M.D., Thomas G.H. (2006). Brush border spectrin is required for early endosome recycling in *Drosophila*. J. Cell Sci..

[90] Apodaca G. (2017). Role of polarity proteins in the generation and organization of apical surface protrusions. Cold Spring Harb. Perspect. Biol..

[91] Hirokawa N., Cheney R.E., Willard M. (1983). Location of a protein of the fodrin-spectrin-TW260/240 family in the mouse intestinal brush border. Cell.

[92] Crawley S.W., Mooseker M.S., Tyska M.J. (2014). Shaping the intestinal brush border. J. Cell Biol..

[93] Danielsen E.M., Hansen G.H. (2016). Small molecule pinocytosis and clathrin-dependent endocytosis at the intestinal brush border: Two separate pathways into the enterocyte. Biochim. Biophys. Acta Biomembr..

[94] Kamal A., Ying Y.-S., Anderson R.G.W. (1998). Annexin VI-mediated loss of spectrin during coated pit budding is coupled to delivery of LDL to lysosomes. J. Cell Biol..

[95] Michaely P., Kamal A., Anderson R.G.W., Bennett V. (1999). A requirement for ankyrin binding to clathrin during coated pit budding. J. Biol. Chem..

[96] Wigge P., Vallis Y., McMahon H.T. (1997). Inhibition of receptor-mediated endocytosis by the amphiphysin SH3 domain. Curr. Biol..

[97] Ghisleni A., Galli C., Monzo P., Ascione F., Fardin M.-A., Scita G., Li Q., Maiuri P., Gauthier N.C. (2020). Complementary mesoscale dynamics of spectrin and acto-myosin shape membrane territories during mechanoresponse. Nat. Commun..

[98] Parton R., Dotti C.G. (1993). Cell biology of neuronal endocytosis. J. Neurosci. Res..

[99] Leshchyns’Ka I., Sytnyk V., Richter M., Andreyeva A., Puchkov D., Schachner M. (2006). The adhesion molecule CHL1 regulates uncoating of clathrin-coated synaptic vesicles. Neuron.

[100] Gong Q., Huntsman C., Ma D. (2007). Membrane trafficking review series: Clathrin-independent internalization and recycling. J. Cell Mol. Med..

[101] Tian N., Leshchyns’Ka I., Welch J.H., Diakowski W., Yang H., Schachner M., Sytnyk V. (2012). Lipid raft-dependent endocytosis of close homolog of adhesion molecule L1 (CHL1) promotes neuritogenesis. J. Biol. Chem..

[102] Puchkov D., Leshchyns’Ka I., Nikonenko A.G., Schachner M., Sytnyk V. (2011). NCAM/spectrin complex disassembly results in PSD perforation and postsynaptic endocytic zone formation. Cereb. Cortex.

[103] Baines A. (2010). Evolution of the spectrin-based membrane skeleton. Transfus. Clin. Biol..

[104] Broderick M., Winder S. (2005). Spectrin, α-actinin, and dystrophin. Adv. Protein Chem..

[105] Letunic I., Khedkar S., Bork P. (2020). SMART: Recent updates, new developments and status in 2020. Nucleic Acids Res..

[106] Jeng R.L., Welch M.D. (2001). Cytoskeleton: Actin and endocytosis—No longer the weakest link. Curr. Biol..

[107] Dragwidge J.M., Van Damme D. (2020). Visualising endocytosis in plants: Past, present, and future. J. Microsc..

[108] Cram W.J. (1980). Pinocytosis in plants. New Phytol..

[109] Fan L., Li R., Pan J., Ding Z., Lin J. (2015). Endocytosis and its regulation in plants. Trends Plant Sci..

[110] Goode B.L., Eskin J.A., Wendland B. (2015). Actin and endocytosis in budding yeast. Genetics.

[111] Kaksonen M., Sun Y., Drubin D.G. (2003). A pathway for association of receptors, adaptors, and actin during endocytic internalization. Cell.

[112] Smythe E., Ayscough K.R. (2006). Actin regulation in endocytosis. J. Cell Sci..

[113] Addario B., Sandblad L., Persson K., Backman L. (2016). Characterisation of *Schizosaccharomyces pombe* α-actinin. PeerJ.

[114] Dejonghe W., Kuenen S., Mylle E., Vasileva M., Keech O., Viotti C., Swerts J., Fendrych M., Ortiz-Morea F.A., Mishev K. (2016). Mitochondrial uncouplers inhibit clathrin-mediated endocytosis largely through cytoplasmic acidification. Nat. Commun..

